# Fear of predators alters herbivore regulation of soil microbial community function

**DOI:** 10.1002/ece3.10207

**Published:** 2023-06-29

**Authors:** Wei Tian, Dror Hawlena, Jordi F. Pagès, Zhiwei Zhong, Deli Wang

**Affiliations:** ^1^ Institute of Grassland Science, Key Laboratory of Vegetation Ecology, Ministry of Education/Jilin Songnen Grassland Ecosystem National Observation and Research Station Northeast Normal University Changchun China; ^2^ Department of Ecology, Evolution, and Behavior, Alexander Silberman Institute of Life Sciences The Hebrew University of Jerusalem Jerusalem Israel; ^3^ Centre d'Estudis Avançats de Blanes (CEAB‐CSIC) Blanes Spain; ^4^ Departament de Biologia Evolutiva, Ecologia i Ciències Ambientals, Facultat de Biologia Universitat de Barcelona Barcelona Spain

**Keywords:** body stoichiometry, enzymatic stoichiometry, enzyme activity, herbivore, predator

## Abstract

Fear of predation can affect important ecosystem processes by altering the prey traits expression that, in turn, regulates the quantity and quality of nutritional inputs to soil. Here, we aimed to assist in bridging a knowledge gap in this cascading chain of events by exploring how risk of spider predation may affect grasshopper prey performances, and the activity of various microbial extracellular enzymes in the soil. Using a mesocosms field‐experiment, we found that grasshoppers threatened by spider predation ate less, grew slower, and had a higher body carbon to nitrogen ratio. Herbivory increased activity of all microbial extracellular enzymes examined, likely due to higher availability of root exudates. Predation risk had no effect on C‐acquiring enzymes but decreased activity of P‐acquiring enzymes. We found contrasting results regarding the effect of predation on the activity of N‐acetyl‐glucosaminidase and leucine arylamidase N‐acquiring enzymes, suggesting that predation risk may alter the composition of N‐inputs to soil. Our work highlighted the importance of soil microbial enzymatic activity as a way to predict how changes in the aboveground food‐web dynamics may alter key ecosystem processes like nutritional‐cycling.

## Graphical abstract

Predation risk reduced herbivore regulation on microbial‐mediated P mineralization. Predation risk decreased the positive effect of herbivores on microbial N limitation. Predation risk did not influence the effect of herbivores on microbial C limitation

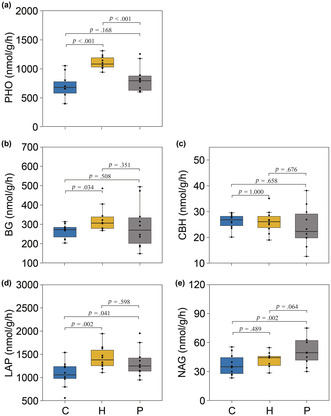

## 1 INTRODUCTION

Predators play a crucial role in regulating ecosystem functioning by inducing trophic cascades (Leroux et al., 2012; Schmitz et al., 2010; Xi et al., 2013). It is widely accepted that the mere risk of predation can have an equal or even greater effect on ecosystem processes than the consumptive effect of predators (Preisser et al., 2005; Schmitz et al., 2010). This is mainly because fear from predators can induce behavioral, morphological, and physiological changes in the expression of prey traits (Sheriff et al., 2020). These changes in prey trophic function may alter key ecosystem processes, including elemental cycling (Hawlena et al., 2012; Leroux et al., 2012; Schmitz et al., 1997). Ample research was focused on how predation risk affects prey traits and consequently regulates ecosystem functioning (Hawlena et al., 2012; Schmitz et al., 2004, 2010), and on how herbivory regulates the soil microbial community function (Huang et al., 2013; Prather et al., 2017). What remains largely unknown is how predation risk regulates the function of soil microbial communities.

Herbivory controls microbial activity by regulating the quantity and nutritional quality of root exudates (Bardgett & Wardle, 2010; Hamilton et al., 2008), and by transforming the recalcitrant plant‐material into easily accessible and nutritionally rich waste materials (Frost & Hunter, 2004; Potthast et al., 2021). Predators can indirectly regulate these pathways by altering herbivores' foraging behavior to prioritize safety over food (Brown & Kotler, 2004), and by favoring food resources that are rich in digestible carbohydrate‐C to fuel preys' heightened energy demands (Hawlena & Schmitz, 2010). Moreover, prey stressed by predators allocate resources from growth and reproduction to sustain their elevated metabolism. These changes may alter preys' body composition, increasing the body Carbon‐to‐Nitrogen (C:N) ratio, and the nutritional composition of prey waste materials to include more N‐rich compounds (Rinehart & Hawlena, 2020). For example, Hawlena et al. (2012) showed that grasshoppers stressed by spider predation have a higher body C:N ratio, and a higher waste‐N content than grasshoppers raised in risk free environments. Consequently, predation risk should affect the quantity and nutritional quality of the nutrient inputs to soil, potentially regulating the microbial community function.

Microorganisms produce extracellular enzymes that break down complex organic compounds in an attempt to fulfill their nutritional demand (Malik et al., 2020). Whenever new organic inputs alleviate microbial nutritional deficiencies, microorganisms respond by decreasing the production of enzymes that degrade complex substrates (Allison & Vitousek, 2005). The strength of these responses, however, depended on the nutritional composition of soil inputs (Chen et al., 2020). As a result, characterizing the activity of soil microbial extracellular enzymes may allow assess in what nutrients limit the microbial community (Gusewell & Freeman, 2005; Sinsabaugh & Shah, 2012).

Our goal was to examine the effect of predation risk on prey‐mediated soil microbial community functions. We reared *Euchorthippus unicolor* grasshoppers in field mesocosms with and without *Lycosid* wolf spider predator, and examined the grasshoppers' feeding performances, body C:N ratio, and growth. We also characterized the extracellular enzymes' activity in the soil. We hypothesized that spider presence may reduce herbivory pressure by grasshoppers, increasing allocation of plant resources to belowground. This may lead to overall higher microbial biomass and higher activity of soil microbial enzymes. Spider presence may decrease soil inputs but alter their nutritional composition to include relatively more N, alleviating the soil microbial N limitation. Predation risk may, thus, increase the ratio between the C and P acquiring enzymes and N acquiring enzymes, leading to higher enzymes C:N and lower N:P ratios.

## 2 MATERIALS AND METHODS

### 2.1 Site description and experiment design

We conducted our field experiment at Jilin Songnen Grassland Ecosystem National Observation and Research Station, Changling, China (44°45′ N, 123°45′ E). The climate in this site is semi‐arid continental and monsoon with average annual precipitation ranging from 280 to 400 mm. This system is dominated by the grass *L. chinensis*, and its main insect herbivore *E. unicolor* grasshopper. Wolf spiders are common predators of grasshoppers in this system.

**FIGURE 1:**
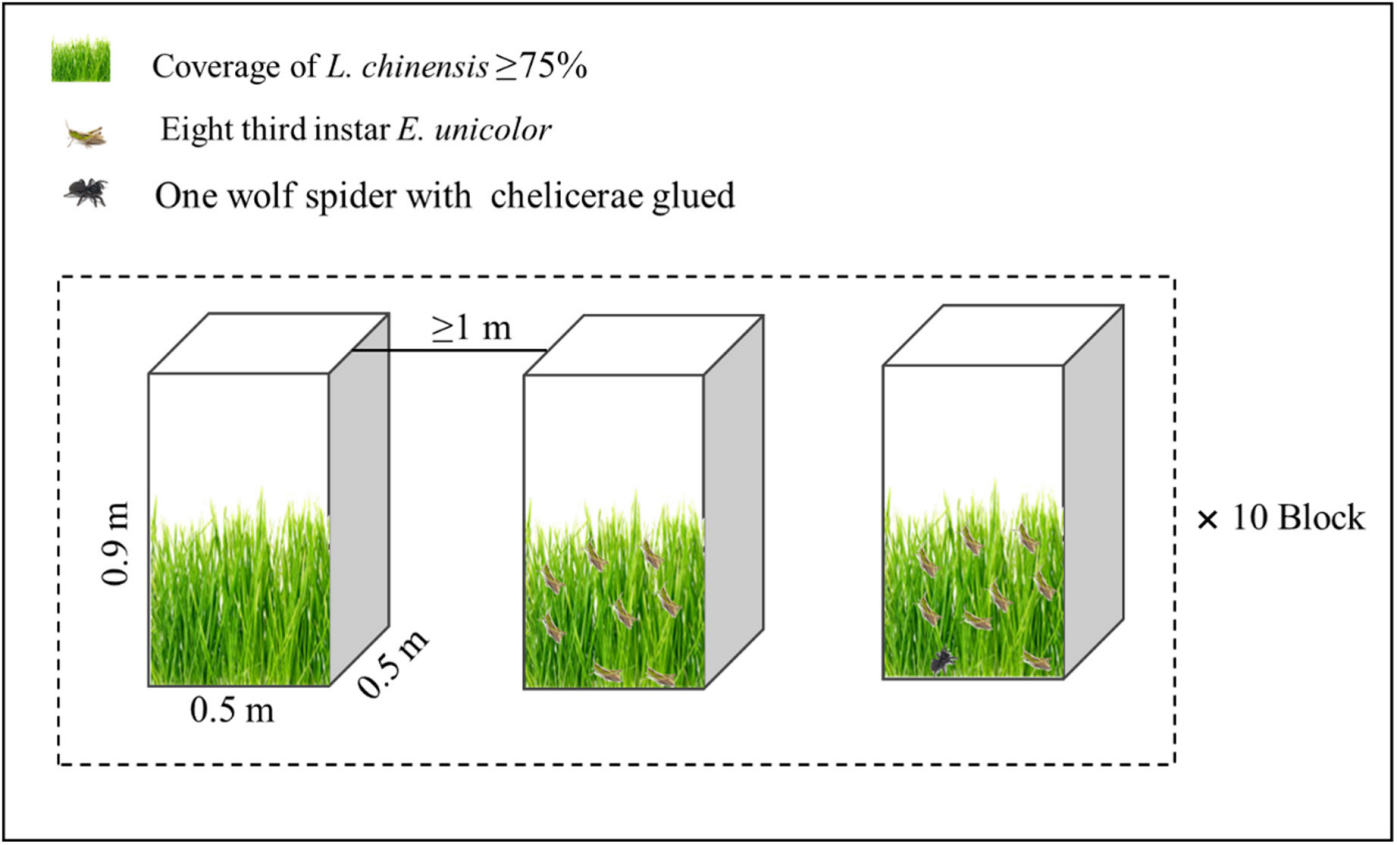
The design of a three‐trophic‐level treatment experiment, containing (i) plants (*Leymus chinensis*), (ii) plants and grasshoppers (*Leymus chinensis* and eight third instar *Euchorthippus unicolor*), and (iii) plants, grasshoppers, and spiders (*Leymus chinensis*, eight third instar *Euchorthippus unicolor* and a treated wolf spider).

We tested how risk of spider predation regulates grasshoppers' trophic function and subsequently soil microorganism enzymatic activity and stoichiometry, by setting a randomized block experiment with three‐trophic‐level treatments: (i) plants (control), (ii) plants and grasshoppers (herbivore), and (iii) plants, grasshoppers, and spiders (predation risk; Figure 1). We constructed 10 blocks of three 0.25‐m^2^‐basal area, 0.9‐m‐high mesocosms covered by 0.8 mm × 1.2 mm mesh size screen. All mesocosms were constructed over naturally growing vegetation of similar composition, in which *L. chinensis* accounted for about 75% of the soil surface coverage. The blocks were at least 10 meters apart. Before stocking the mesocosms, we removed all naturally occurring invertebrates using a vacuum cleaner. Grasshopper density in this field site reaches a maximum of 20–30 per m^2^ (Zhong et al., 2014). Thus, we stocked each mesocosm with eight third instar grasshoppers and added one wolf spider to each predation mesocosm. We glued the spider chelicerae with quick‐drying glue to prevent them from preying upon grasshoppers (Hawlena & Schmitz, 2010). Previous work in our own study system, and elsewhere have shown that spiders with glued chelicerae can remain active for more than 2 months (Barton, 2010; Hawlena et al., 2012). Our experiment lasted from 10 August to 20 September 2020.

### 2.2 Grasshopper feeding frequency, survival rate, growth rate, and body C:N

We conducted feeding measurements 7 days after stocking the mesocosms, to allow grasshoppers and spiders to settle within the mesocosms. We randomly selected a third‐instar *E. unicolor* from each mesocosm, and painted a mark on its thorax to enable focal observations. The observer recorded the times of grasshopper feeding behavior (eating foliage without retracting the heat from the plants) in their most active period (07:00–09:00, 11:00–13:00, and 15:00–17:00), for a total of 6 h, and calculated total number of feeding time in 6 h as feeding frequency (Zhong et al., 2017). All grasshoppers were weighed at the beginning and end of the experiment, and the grasshopper growth‐rate was calculated as the difference between the final and initial wet weight divided by the number of days elapsed. Grasshopper survival rate was calculated as the final density divided by the initial density in each mesocosm. At the end of the experiment, we starved all surviving grasshoppers for 24 h to allow gut evacuation, and then freeze‐dried and homogenized their carcasses using MM400 ball mill (Retsch GmbH Rheinische Strabe 3642781). We used C:N:H elemental analyzer to quantify grasshoppers' body C and N content (Vario EL cube; Elementar).

### 2.3 Soil sampling and analysis of soil enzymatic activity

In each mesocosm, we collected five soil subsamples from 0 to 10 cm depth. The samples were thoroughly mixed to make a composite homogenous soil sample per mesocosm. We stored all samples at 4°C for 14 days and then analyzed them for microbial extracellular enzymatic activity. We measured the activity of one P acquiring enzyme (acid phosphatase, PHO), two C acquiring enzymes (β‐d‐Glucoside, BG; Cellobiohydrolase, CBH), and two N acquiring enzymes (N‐Acetyl‐glucosaminidase, NAG; Leucine arylamidase, LAP), following the method described by DeForest (2009). In addition, the enzymatic stoichiometries of (BG + CBH):(NAG + LAP), (BG + CBH):PHO, and (NAG + LAP):PHO were calculated as the indicators for microbial C versus N, C versus P, and N versus P demand, respectively. Higher microbial C:N and C:P enzymatic ratios mean higher microbial C demand relative to N and P demand respectively. Higher microbial N:P enzymatic ratio represents higher microbial N demand relative to P demand (Luo et al., 2017; Waring et al., 2014).

### 2.4 Data analyses

We used linear mixed‐effects models in the package “lme4” (Bates et al., 2014) to test how predation risk affects grasshopper performances (grasshopper survival rate, growth rate, body C:N, and feeding frequency). The number of trophic levels (plant only, herbivore treatment and predation risk treatment) in each mesocosm was set as a fixed effect factor and blocks were treated as a random factor. We used Poisson distribution to explore consequences of predation on grasshoppers feeding. We used a similar approach to explore how predation risk affects soil enzyme activity and stoichiometries (PHO, BG, CBH, LAP, NAG, enzyme C:P, C:N, and N:P). All response variables were tested for normality and homogeneity of variance and log transformation was conducted when needed. The statistical analyses were performed using R 4.1.2 software (R Development Core Team, 2019).

## 3 RESULTS

### 3.1 The effects of predation risk on grasshopper survival rate, growth rate, body C:N and feeding frequency

**FIGURE 2:**
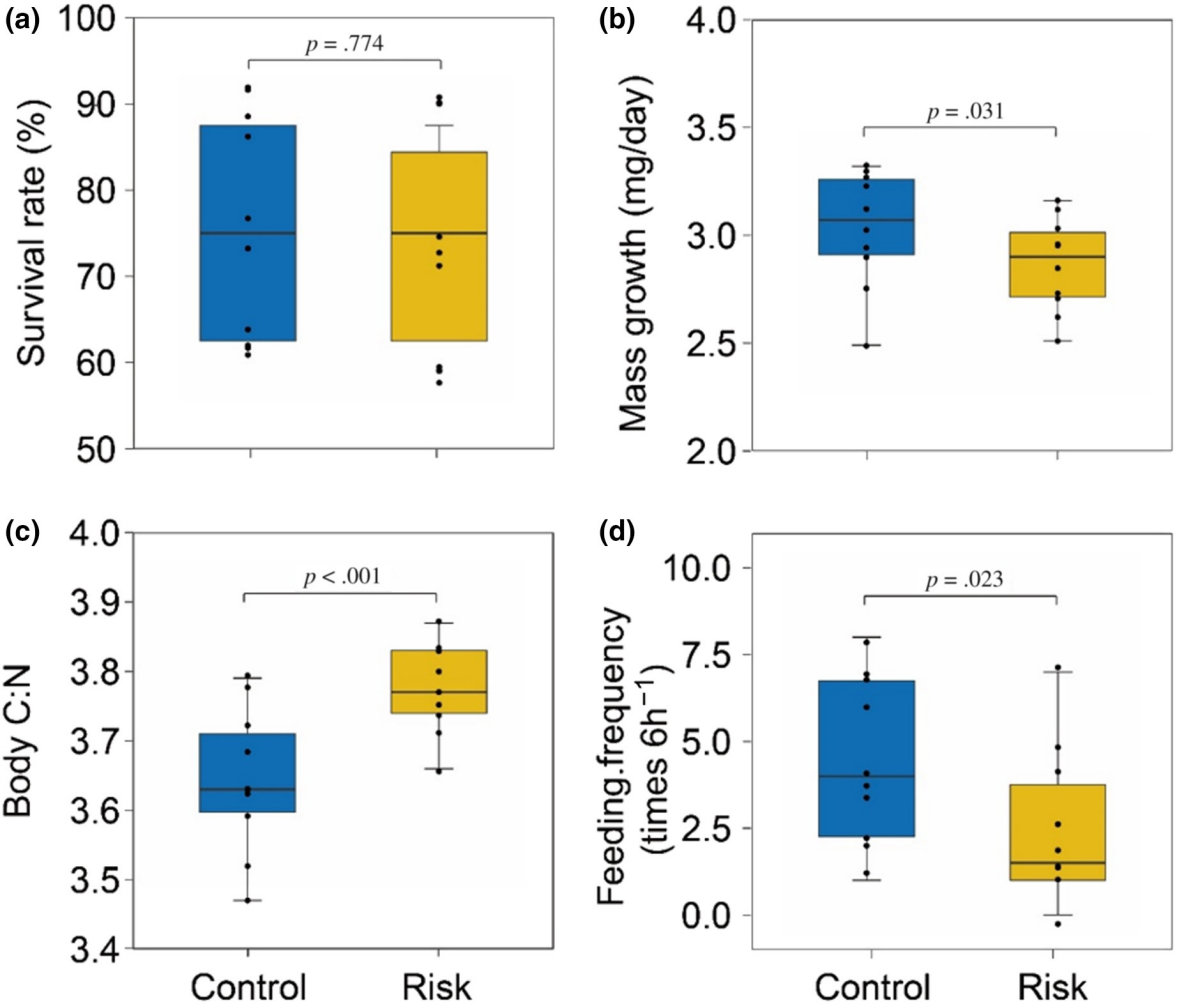
Effects of predation risk on the performance of the grasshopper *Euchorthippus unicolor*. (a) Survival rate, (b) mass growth rate, (c) body C:N, and (d) feeding frequency. Control indicates herbivore treatment. Risk represents predation risk treatment. The lines in the boxes are median values, and the edges of the boxes represent 25% and 75% percentiles, respectively, and the black dots represent the value in each block. Results of the Tukey test used for pairwise comparisons among treatments (*p* values) are displayed above bars.

Predation risk did not affect the survival rate of grasshoppers compared to herbivore treatment (*χ* ^2^ = 0.08, df = 1, *p* = .774; Figure 2a). Grasshoppers under elevated risk of predation grew slower (*χ* ^2^ = 4.65, df = 1, *p* = .031; Figure 2b) and reduced feeding frequency (*χ* ^2^ = 5.17, df = 1, *p* = .023; Figure 2d), but had higher body C:N ratio (*χ* ^2^ = 25.60, df = 1, *p* < .001; Figure 2c) in comparison to the no‐risk treatment.

### 3.2 Effects of predation risk on soil microbial enzymatic activities and stoichiometry

**FIGURE 3:**
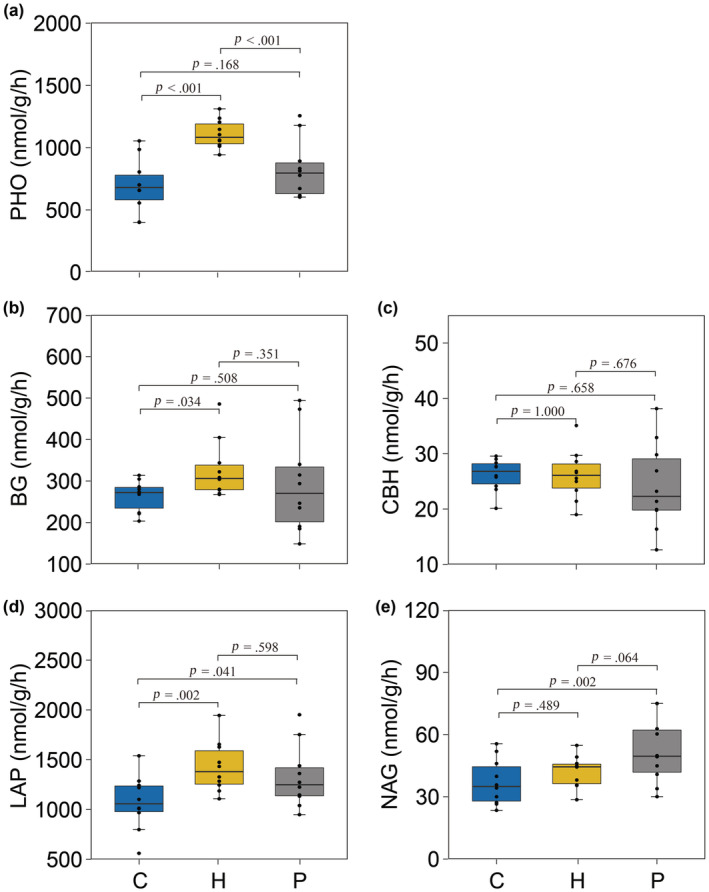
Effects of herbivores and predation risk on soil microbial enzymatic activities. (a) Leucine arylamidase (LAP), (b) N‐acetyl‐glucosaminidase (NAG), (c) β‐d‐glucoside (BG), (d) cellobiohydrolase (CBH), and (e) acid phosphatase (PHO). C: control treatment, H: herbivore treatment, P: both herbivore and predation risk treatment. The lines in the boxes are median values, and the edges of the boxes represent 25% and 75% percentiles, respectively, and the black dots represent value in each block. Results of the Tukey test used for pairwise comparisons among treatments (*p* values) are displayed above bars.

Soil microbial PHO, BG, LAP, and NAG activity differed between experimental treatments, but CBH activity was similar across all treatments (Table 1 and Figure 3). Specifically, the soil PHO, BG, and LAP activities were significantly higher in the grasshopper only treatment than in the control no‐grasshopper treatment (PHO: *p* < .001, BG: *p* = .034, LAP: *p* = .002; Figure 3a,b,d). Grasshopper reared under elevated risk of predation reduced soil PHO enzymatic activity and did not influence soil BG and LAP activities compared to the no risk treatment (PHO: *p* < .001, BG: *p* = .351, LAP: *p* = .598; Figure 3a,b,d). NAG activity was similar between the plants only and grasshopper treatments (*p* = .489; Figure 3e), but was significantly higher in the predation risk treatment than in the grasshopper only control (*p* = .064; Figure 3e).

**TABLE 1:** Linear mixed‐effects model results show the effects of treatments on soil enzymatic activities and stoichiometries.

	*χ* ^2^	df	*p*
PHO	33.57	2	<.001
BG	6.22	2	.045
CBH	0.98	2	.613
LAP	12.20	2	.002
NAG	11.85	2	.003
Enzyme C:N	3.69	2	.158
Enzyme C:P	16.35	2	<.001
Enzyme N:P	15.14	2	<.001

Abbreviations: BG, β‐d‐Glucoside; CBH, Cellobiohydrolase; LAP, Leucine arylamidase; NAG, N‐Acetyl‐glucosaminidase; PHO, acid phosphatase.

**FIGURE 4:**
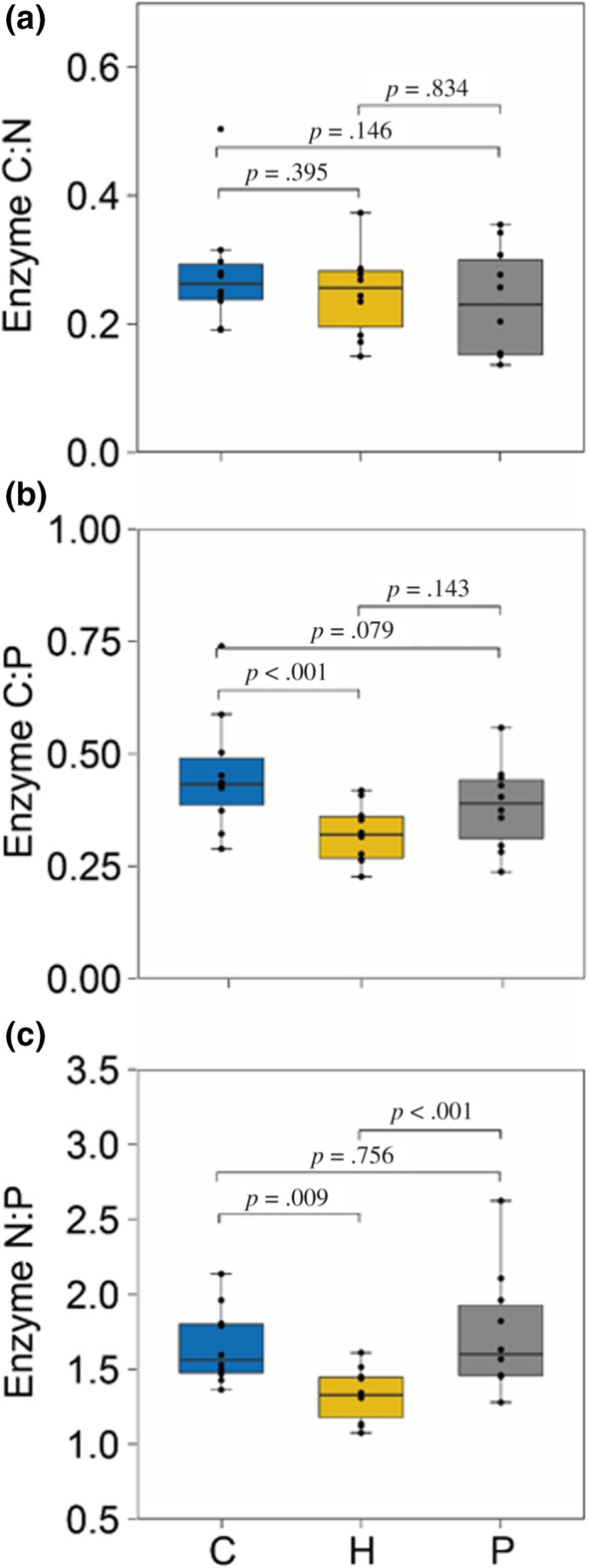
Effects of insect herbivores and predation risk treatments on soil microbial carbon and nutrient limitation. (a) Microbial C:N enzymatic ratio, (b) microbial C:P enzymatic ratio, and (c) microbial N:P enzymatic ratio. C: control treatment, H: herbivore treatment, P: both herbivore and predation risk treatment. The microbical C:N enzymatic ratio represents (BG + CBH):(NAG + LAP), microbial C:P enzymatic ratio indicates (BG + CBH):PHO, and microbial N:P enzymatic ratio means (NAG + LAP):PHO. The lines in the boxes are median values, and the edges of the boxes represent 25% and 75% percentiles, respectively, and the black dots represent value in each block. Results of the Tukey test used for pairwise comparisons among treatments (*p* values) are displayed above bars.

Microbial C:P and N:P enzymatic ratios differed between treatments but microbial C:N enzymatic ratio was similar across treatments (Table 1 and Figure 4). Specially, the herbivore treatment reduced microbial C:P enzymatic ratio compared to the no–grasshoppers control treatment (*p* < .001; Figure 4b) but did not differ between the grasshoppers only and predation risk treatments (*p* = .143; Figure 4b). Furthermore, the microbial N:P enzymatic ratio was significantly lower in the herbivore treatments compare to control or predation risk treatments (herbivore vs. control: *p* = .009, herbivore vs. predation risk: *p* < .001; Figure 4c).

## 4 DISCUSSION

Predation risk plays a key role in regulating ecosystem nutrient dynamics by inducing defensive expressions of prey traits that alter the quantity and nutritional quality of soil inputs (Hawlena et al., 2012; Schmitz et al., 2010; Strickland et al., 2013). We aimed to bridge a key knowledge‐gap in this cascading chain of events by exploring how predation risk affects the soil microbial community function, focusing on the activity of extracellular enzymes. Using mesocosms field experiment, we found that grasshoppers threatened by spider predation ate less, grew slower, and had higher body C:N, coinciding with previous work (Barton, 2010; Hawlena & Schmitz, 2010). We also found that enzyme activity was lower in the plant only control in comparison to when grasshoppers were present, supporting our hypothesis that herbivory should enhance soil inputs. Contrary to our expectations, we found no differences in enzyme activity between the risk and risk free treatments except for P acquiring enzymes. PHO activity was lower in the presence of spiders than in their absence. Activity of the N‐ acquiring enzyme LAP was higher in risky environments compared to the plant only control, suggesting lower N inputs in risky environments. Exploration of the stoichiometric relationships between the activities of extracellular enzymes supported these general insights as C:P ratio was lower in the grasshoppers only treatment compared to the plants only control, and N:P ratio in the grasshoppers only treatment was lower compared to the elevated risk and plant only treatments.

Herbivory increases carbon allocation to below‐ground organs, and in turn increases root exudation (Holland et al., 1996). Root exudates typically include organic acids, sugars, and amino acids (Martin & Marschner, 1988). Thus, herbivore‐induced increases in root exudates can support higher microbial biomass and consequently elevated extracellular enzyme activity (Bardgett & Wardle, 2010; Hamilton et al., 2008; Hamilton & Frank, 2001). Our results coincided with this expectation. We found that herbivorous grasshoppers significantly increased microbial P (PHO), C (BG), and N (LAP) acquiring enzymatic activities (Figure 3a,b,d). Our results also suggested that not only the quantity but also the nutritional composition of the soil inputs were affected by herbivory. This is because the C:P and N:P ratios of the extracellular acquiring enzymes was lower in the grasshopper only treatment compared to the plant only control (Figure 4b,c), implying that herbivory lessened P relatively to C and N inputs to soil.

Prey respond to elevated risk of predation by constraining their foraging activity and by inducing physiological stress responses that alter their dietary demands (Hawlena et al., 2011; Hawlena & Schmitz, 2010; Schmitz et al., 1997). Our experimental results coincided with this prediction showing that grasshoppers reared with predators reduced feeding frequency and growth (Figure 2d), providing an indirect evidence for lower herbivory pressure. Lower herbivory should lead to lower release of root exudates and consequently to lower soil microbial biomass and function (Huang et al., 2013). Thus, we hypothesized that predation risk should decrease activity of microbial extracellular enzymes in the soil. Our results rejected this hypothesis except for P (PHO) acquiring enzyme. In all other measurements, we found no indirect effect of predation risk on the activity of soil microbial enzymes that was different from that found in the grasshopper treatment (Figure 3a–e). Our results could not allow us to identify the exact mechanism responsible for this predator induced change, yet provided clear evidence that predation can indirectly decrease soil microbial P demand.

Predation may affect soil communities not only by regulating the excretion of root exudates but also by controlling the quantity and nutritional quality of animal derived inputs to soil (Hawlena & Zaguri, 2016). For example, grasshoppers stressed by risk of spider predation produced feces that included 40% more N than conspecifics that experienced no risk of predation and demonstrated increase in body C:N ratio as was found in this study (Hawlena & Schmitz, 2010). Such changes in prey body composition led to substantially slower plant litter decomposition (Hawlena et al., 2012). We found that predation risk did not influence LAP N‐acquiring enzymes activity compared to that in herbivore treatment (Figure 3d). Examination of the NAG N‐acquiring enzyme activity revealed different results that predation showed higher activity of the NAG N‐acquiring enzymes compared to the grasshopper only treatment (Figure 3e). The stoichiometric decomposition theory suggests that when N is limiting, an input of a small amount of N will increase microbial N‐acquiring enzymatic activity in attempt fulfill the needs (Sterner & Elser, 2003). Thus, our findings implied that risk of predation may limit only certain N‐inputs to soil while maintaining others. We found support to this interpretation when examining the stoichiometric relationship between the extracellular enzyme activities. Predation risk increased soil microbial N:P enzymatic ratio compared to the herbivore only treatment (Figure 4c), suggesting that predation promotes the microbial N demand relative to P demand. Consequently, we demonstrated that despite the expected increase in fecal N content of grasshoppers stressed by predators the microbial community still experienced N‐limitation. Future work should examine the different prey derived inputs in isolation to reveal the detailed mechanistic pathway by which predation regulates soil communities.

Our findings showed that predation risk had no effect on CBH and BG enzymatic activities. Interestingly, we also found that microbial C:N and C:P enzymatic ratios in the predation risk treatment were similar to the treatment of herbivores (Figure 4a,b), showing that predation risk did not influence soil microbial C limitation. Two possible mechanisms may explain this finding: (1) Compared with herbivore treatment, increases of N‐acquiring enzymes (NAG) might be catalyzing the terminal reaction of chitin and peptidoglycan degradation, which both release labile N and C (Luo et al., 2017), contributing to microbial demand for carbon, and (2) predation risk might decrease soil microbial biomass through reducing herbivore‐mediated plant root exudation (Nat Holland, 1995; Schmitz et al., 1997), and lower microbial biomass has been shown to have comparatively lower needs for carbon (Allison et al., 2010). These also help explain the weak variation of BG and CBH activity between herbivores only and predation risk treatment.

In summary, our work reveled how structural changes in an aboveground food‐web may affect the microbial extracellular activity belowground, while emphasizing the effect of predation risk. These findings also contribute whole new mechanistic insights to the growing recognition that trophic interactions may play a key role in understanding the links between above‐ and belowground food webs. As such, our work indicates that soil microbial enzymatic activity can link food‐web interactions aboveground and microbial responses belowground to better explain ecosystem nutrient dynamics.

## AUTHOR CONTRIBUTIONS

**Wei Tian:** Conceptualization (lead); formal analysis (lead); methodology (supporting); visualization (lead); writing – original draft (lead); writing – review and editing (lead). **Dror Hawlena:** Writing – review and editing (equal). **Jordi F. Pagès:** Writing – review and editing (equal). **Zhiwei Zhong:** Writing – review and editing (equal). **Deli Wang:** Conceptualization (lead); writing – review and editing (lead).

## CONFLICT OF INTEREST STATEMENT

All authors certify that they do not have any conflict of interest to disclose.

## Data Availability

The data that support the findings of this study are openly available in Dryad at doi:10.5061/dryad.x3ffbg7np.
